# Draft genome sequence of *Anoxybacillus flavithermus* KU2-6-11 isolated from hot-spring in Uzon caldera (Kamchatka, Russia)

**DOI:** 10.1016/j.dib.2017.11.095

**Published:** 2017-12-06

**Authors:** Aleksey S. Rozanov, Anton V. Korzhuk, Alla V. Bryanskaya, Sergey E. Peltek

**Affiliations:** Federal Research Center "Institute of Cytology and Genetics of the Siberian Branch of the RAS", Novosibirsk, Russia

**Keywords:** Thermophiles, Hot spring, Uzon caldera, Kamchatka, *Anoxybacillus flavithermus*, Illumina MiSeq, Whole genome sequence

## Abstract

The *Anoxybacillus flavithermus* KU2-6-11 was isolated from sediments of a nameless hot spring. The hot spring is located in Uzon caldera (Kamchatka, Russia). The sequenced and annotated genome is 2,646,305 bp and encodes 2787genes. The draft genome sequence of the *Anoxybacillus flavithermus* KU2-6-11 has been deposited at DDBJ/EMBL/GenBank under the accession PEDM01000000 and the sequences could be found at the site https://www.ncbi.nlm.nih.gov/nuccore/PEDM01000000.

**Specifications**

**Table:** 

Subject area	*Biology*
More specific subject area	*Microbiology, Genomics*
Type of data	Genomic sequence, gene prediction of *Anoxybacillus flavithermus*
How data was acquired	Shotgun whole-genome DNA sequencing using MiSeq platform (Illumina) at the "Molecular and cellular biology" facility at IMCB SB RAS
Data format	Draft genome assembly, and gene prediction
Experimental factors	DNA was extracted from *Anoxybacillus flavithermus* KU2-6-11
Experimental features	Whole genome shotgun sequencing followed by genome assembly and gene description
Data source location	*A. flavithermus* KU2-6-11 was isolated from a cyanobacteria mat from nameless hot spring (65 °C) in Uzon Caldera (Kamchatka, Russia), 10 m from Zavarzin hot spring. (54^о^ 29.883' N; 160^о^ 00.874' E)
Data accessibility	The draft genome sequence for *Anoxybacillus flavithermus* strain KU2-6-11 has been deposited in DDBJ/EMBL/Genbank under the accession no. PEDM01000000. The 74 contigs have been deposited under accession no. PEDM01000001–PEDM01000074.
	https://www.ncbi.nlm.nih.gov/nuccore/PEDM01000000

**Value of the data**

• Genome *Anoxybacillus flavithermus* consist genes important for biotechnology.
• Draft genome assembly of *Anoxybacillus flavithermus,* from poorly studied region, will increase the knowledge of the biochemical process of thermophilic communities and create an opportunity for comparative studies with other bacteria.

## 1 Data

Anoxybacillus was defined as a new genus in 2000, the first type strain was *A. pushchinoensis* [1]. In this work, Bacillus flavithermus was reclassified as Anoxybacillus flavithermus [1]. The members of this genus are alkaliphilic, moderately thermophilic, spore-forming, Gram-positive bacteria belonging to the order of Bacillales. Present time the genus Anoxybacillus include more 15 species [1], [2], [3], [4], [5]. Species of this genus are widely distributed in geothermal springs [1], [6]. In Russia they were detected in geothermal springs of Baikal rift zone [7], in the Geyser valley, Kamchatka [8].

*Anoxybacillus flavithermus* strain KU2-6-11 was isolated from cyanobacterial mat from nameless hot spring (65 °C) in Uzon Caldera (Kamchatka, Russia, 54^о^ 29.883' N; 160^о^ 00.874' E), 10 m from Zavarzin hot spring [9]. The hot spring has diameter of less than 1 m. The threads of sulfur bacteria developing on the edge of the funnel and outflow of the stream by, replaced by cyano and algo-bacterial communities. We isolated the strain for Collection of biotechnological microorganisms as a source of novel promising objects for biotechnology and bioengineering of Federal Research Center "Institute of Cytology and Genetics of the Siberian Branch of the RAS".

The draft genome sequence for *Anoxybacillus flavithermus* strain KU2-6-11 has been deposited in DDBJ/EMBL/Genbank under the accession no. PEDM01000000. The 74 contigs have been deposited under accession no. PEDM01000001–PEDM01000074.

## 2 Experimental design, materials, and methods

### 2.1 Library

• Strategy: Whole-genome DNA sequencing.
• Taxon: *Anoxybacillus flavithermus*.
• Sample details: The strain was isolated in 2011 by Alla V. Bryanskaya and stored at Collection of biotechnological microorganisms as a source of novel promising objects for biotechnology and bioengineering of Federal Research Center "Institute of Cytology and Genetics of the Siberian Branch of the RAS".
• Location: Uzon Caldera (Kamchatka, Russia), 10 m from Zavarzin hot spring. (54^о^ 29.883' N; 160^о^ 00.874' E).
• Sample handling: culture was cultivated in liquid medium containing 1% trypton, 0.5% yeast extract, and 1%of NaCl. DNA was extracted from 8 ml using the DNA Purification Kit (Fermentas).
• Library: Paired-end 75 bp reads.

### 2.2 DNA isolation and sequencing

*A.flavithermus* KU2-6-11 culture was cultivated in liquid medium containing 1% trypton, 0.5% yeast extract, and 1%of NaCl. Eight ml of cell culture were pelleted by centrifugation and resuspended in 75 µl of H_2_O by intense pipetting. DNA was isolated using the DNA Purification Kit (Fermentas). Nebnext® ultra**™** II DNA library prep kit for Illumina (New England Biolabs, USA) were used to create libraries for genome sequencing. Genome sequencing was performed on MiSeq (Illumina), using MiSeq Reagent Kit v3 (150-cycle) (Illumina, USA) in the "Molecular and cellular biology" facility at IMCB SB RAS.

### 2.3 Data processing

*De novo* assembly of short reads into contigs was performed using SPAdes v. 3.10.1 [10]. Contigs shorter than 1000 bp were deleted. A total of 74 contigs yielded a genome sequence 2,652,077 bp long, and the G + C content is 41.48%. ORF prediction and automatic annotation was performed using NCBI PGAAP (http://www.ncbi.nlm.nih.gov/genome/annotation_prok). The complete genome sequence contained 2787 genes, 2673 CDS, rRNAs ( 5 - 5S, 1 - 16S, 6 - 23S), 29 tRNAs, 4 ncRNA.

## References

[1] Pikuta E., Lysenko A., Chuvilskaya N., Mendrock U., Hippe H., Suzina N., Nikitin D., Osipov G., Laurinavichius K. (2000). Anoxybacillus pushchinensis gen. nov., sp. nov., a novel anaerobic, alkaliphilic, moderately thermophilic bacterium from manure, and description of Anoxybacillus flavithermus comb. nov. Int. J. Syst. Evol. Microbiol..

[2] Dulger S., Demirbag Z., Belduz A.O. (2004). Anoxybacillus ayderensis sp. nov. and Anoxybacillus kestanbolensis sp. nov. Int. J. Syst. Evol. Microbiol..

[3] Atanassova M., Derekova A., Mandeva R., Sjoholm C., Kambourova M. (2008). Anoxybacillus bogrovensis sp. nov., a novel thermophilic bacterium isolated from a hot spring in Dolni Bogrov, Bulgaria. Int. J. Syst. Evol. Microbiol..

[4] Zhang C.-M., Huang X.-W., Pan W.-Z., Zhang J., Wei K.-B., Klenk H.-P., Tang S.-K., Li W.-J., Zhang K. -q (2011). Anoxybacillus tengchongensis sp. nov. and Anoxybacillus eryuanensis sp. nov., facultatively anaerobic, alkalitolerant bacteria from hot springs. Int. J. Syst. Evol. Microbiol..

[5] Cihan A.C., Cokmus C., Koc M., Ozcan B. (2014). Anoxybacillus calidus sp. nov., a thermophilic bacterium isolated from soil near a thermal power plant. Int. J. Syst. Evol. Microbiol..

[6] Saw J.H., Mountain B.W., Feng L., Omelchenko M.V., Hou S., Saito J.A., Stott M.B., Li D., Zhao G., Wu J., Galperin M.Y., Koonin E.V., Makarova K.S., Wolf Y.I., Rigden D.J., Dunfield P.F., Wang L., Alam M. (2008). Encapsulated in silica: genome, proteome and physiology of the thermophilic bacterium Anoxybacillus flavithermus WK1. Genome Biol..

[7] Rozanov A.S., Bryanskaya A.V., Kotenko A.V., Malup T.K., Peltek S.E. (2014). Draft Genome Sequence of Anoxybacillus flavithermus Strain 25, Isolated from the Garga Hot Spring in the Barguzin Valley, Baikal Region, Russian Federation. Genome Announc..

[8] Kevbrin V.V., Zengler K., Lysenko A.M., Wiegel J. (2005). Anoxybacillus kamchatkensis sp. nov., a novel thermophilic facultative aerobic bacterium with a broad pH optimum from the Geyser valley, Kamchatka. Extremophiles.

[9] Rozanov A.S., Bryanskaya A.V., Malup T.K., Meshcheryakova I.A., Lazareva E.V., Taran O.P., Ivanisenko T.V., Ivanisenko V.A., Zhmodik S.M., Kolchanov N.A., Peltek S.E. (2014). Molecular analysis of the benthos microbial community in Zavarzin thermal spring (Uzon Caldera, Kamchatka, Russia). BMC Genom..

[10] Bankevich A., Nurk S., Antipov D., Gurevich A.A., Dvorkin M., Kulikov A.S., Lesin V.M., Nikolenko S.I., Pham S., Prjibelski A.D., Pyshkin A.V., Sirotkin A.V., Vyahhi N., Tesler G., Alekseyev M.A., Pevzner P.A. (2012). SPAdes: a new genome assembly algorithm and its applications to single-cell sequencing. J. Comput. Biol..

