# Supplementary material for: Draft genome sequence of Anoxybacillus flavithermus KU2-6-11 isolated from hot-spring in Uzon caldera (Kamchatka, Russia)
Source: Data Brief. 2017 Dec 6;16:758–61. doi: 10.1016/j.dib.2017.11.095 (PMC5738195; doi:10.1016/j.dib.2017.11.095)
Supplement: Supplementary file 1 — Supplementary material [file mmc1.docx]

**Conflict of interest statement**

The authors declare that they have no conflict of interest.
